# How Can Technology Improve Burn Wound Care: A Review of Wound Imaging Technologies and Their Application in Burns—UK Experience

**DOI:** 10.3390/diagnostics15172277

**Published:** 2025-09-08

**Authors:** Nawras Farhan, Zakariya Hassan, Mohammad Al Mahdi Ali, Zaid Alqalaf, Roeya E. Rasul, Steven Jeffery

**Affiliations:** Queen Elizabeth Hospital Birmingham, University Hospital Birmingham NHS Trust, Mindelsohn Way, Birmingham B15 2GW, UK

**Keywords:** burns wounds, diagnostic imaging, devices, technology advances, wound healing, LDI, MolecuLight i:X

## Abstract

Burn wounds are complex injuries that require timely and accurate assessment to guide treatment decisions and improve healing outcomes. Traditional clinical evaluations are largely subjective, often leading to delays in intervention and increased risk of complications. Imaging technologies have emerged as valuable tools that enhance diagnostic accuracy and enable objective, real-time assessment of wound characteristics. This review aims to evaluate the range of imaging modalities currently applied in burn wound care and assess their clinical relevance, diagnostic accuracy, and cost-effectiveness. It explores how these technologies address key challenges in wound evaluation, particularly related to burn depth, perfusion status, bacterial burden, and healing potential. A comprehensive narrative review was conducted, drawing on peer-reviewed journal articles, NICE innovation briefings, and clinical trial data. The databases searched included PubMed, Ovid MEDLINE, and the Cochrane Library. Imaging modalities examined include Laser Doppler Imaging (LDI), Fluorescence Imaging (FI), Near-Infrared Spectroscopy (NIR), Hyperspectral Imaging, Spatial Frequency Domain Imaging (SFDI), and digital wound measurement systems. The clinical application and integration of these modalities in UK clinical practice were also explored. Each modality demonstrated unique clinical benefits. LDI was effective in assessing burn depth and perfusion, improving surgical planning, and reducing unnecessary procedures. FI, particularly the MolecuLight i:X device (MolecuLight Inc., Toronto, ON, Canada), accurately identified bacterial burden and guided targeted interventions. NIR and Hyperspectral Imaging provided insights into tissue oxygenation and viability, while SFDI enabled early detection of infection and vascular compromise. Digital measurement tools offered accurate, non-contact assessment and supported telemedicine use. NICE recognized both LDI and MolecuLight as valuable tools with the potential to improve outcomes and reduce healthcare costs. Imaging technologies significantly improve the precision and efficiency of burn wound care. Their ability to offer objective, non-invasive diagnostics enhances clinical decision-making. Future research should focus on broader validation and integration into clinical guidelines to ensure widespread adoption.

## 1. Introduction

Burn wounds are a significant public health concern in the United Kingdom, markedly impacting healthcare utilization, patient outcomes, and economic burden. An estimated 250,000 burn cases occur annually, with approximately 112,000 attendances to accident and emergency departments [1]. Burns account for roughly 1% of emergency room workload and 0.014% of hospitalizations [2]. Pediatric burns are particularly notable, with incidence rates of 16.1 per 10,000 in primary care and 35.1 per 10,000 in emergency settings [3]. Hospital admissions for burn injuries have shown an upward trend over time, influenced by factors such as bed availability [4].

Economically, the direct cost of burn treatment was approximately GBP 89.6 million in 2012–2013, escalating to multi-billion-pound figures when combined with broader wound care expenses [5,6]. Major cost contributors include hospitalization, surgical interventions, outpatient visits, and community nursing [7,8,9]. Cost-effectiveness assessments—for instance, studies on silver dressings for pediatric burns—indicate potential resource efficiencies [10]. However, healthcare reimbursement rates frequently lag behind actual expenditure, imposing financial strain on providers, particularly specialist burn units [8].

Indirect costs, such as lost productivity, unpaid care provision, and out-of-pocket expenses, remain under-reported, though evidence suggests that they comprise a significant portion of the societal burden [11,12]. Long-term impacts such as chronic wounds, disability, and ongoing care requirements further exacerbate economic ramifications, underscoring the importance of longitudinal data for accurate burden assessment [13,14,15].

Contemporary burn management focuses on early diagnosis, prevention of complications, and expedited healing. However, burn wounds continue to pose challenges due to elevated risks of infection, pain, and scar formation—which collectively diminish functional recovery and quality of life [16,17]. Infections are particularly perilous for burn patients, given compromised skin barrier function and immunosuppression [18,19]. Scar sequelae, including hypertrophic and keloid formation, present aesthetic and functional complications that demand early intervention [20,21]. The combination of prolonged hospitalization, psychological distress, and economic impact further emphasizes the critical need for effective burn management strategies [22,23].

Despite these challenges, advances in understanding burn pathophysiology and in diagnostic and therapeutic technologies are creating new opportunities. While these innovations complicate the standardization of care, they also enable research-driven improvements to clinical practice and patient outcomes.

## 2. Innovation

The role of imaging technologies in burn wound care has grown significantly. These modalities increasingly supplement traditional clinical assessment, with some still emerging and others already demonstrating efficacy and potential cost savings.

Burn size and depth have historically been judged via visual inspection and manual measurement. Yet, essential physiological attributes—such as wound architecture, tissue perfusion, and microbial colonization—are not accurately assessed using such methods.

Emerging imaging technologies now allow objective estimations and predictions of healing trajectories. They enable precise wound measurement, perfusion mapping, and bacterial localization—collectively guiding assessments of burn severity and healing potential. Critically, these tools support fast, non-invasive monitoring of wound progression, reducing reliance on subjective evaluation post-treatment.

## 3. Clinical Problem Addressed

This review focused on challenging burn wound care problems, including objective, early assessment and management of burn wounds, particularly in evaluating wound depth, infection risk, and healing trajectory. Traditional assessment methods are often inadequate, leading to delayed treatment and poorer outcomes. Imaging technologies bridge this gap by offering real-time, non-invasive, and quantifiable assessments that directly inform treatment decisions.

## 4. Materials and Methods

A systematic review was conducted in accordance with PRISMA guidelines to evaluate the clinical utility of imaging technologies in burn wound care. Comprehensive searches were performed across databases including PubMed, Ovid MEDLINE, and the Cochrane Library, using a combination of keywords such as “burn imaging”, “burn wound assessment/management”, “diagnostic technology”, “fluorescence imaging burns”, “laser Doppler burns”, and “NIR wound imaging”, “Machine Learning burns”, and “AI-assisted burns”. The inclusion criteria focused on peer-reviewed, English-language studies published between January 2000 and January 2025, with direct clinical relevance to burn care. Studies were excluded if they were non-burn-related, animal-only, or limited to conference abstracts. A total of 245 studies were retrieved from the search and reviewed, and 77 pieces of evidence were included in this review, including clinical trials, NICE innovation briefings, systematic reviews, and observational studies. The review synthesized evidence on Laser Doppler Imaging, Fluorescence Imaging, Near-Infrared Spectroscopy, Hyperspectral Imaging, and Digital Wound Measurement, with particular attention to clinical applicability, outcome reporting, cost-effectiveness, and alignment with UK-based guidelines and healthcare practices. This approach ensures a focused and evidence-driven evaluation of imaging modalities in contemporary burn wound management.

## 5. Results

The application of advanced imaging technologies significantly improved the non-invasive, objective assessment of burn wounds as per this review. Fluorescence imaging techniques demonstrated high accuracy in detecting bacterial burden while enabling real-time wound measurement. Laser Doppler Imaging (LDI) proved effective in assessing tissue perfusion and determining burn depth, enhancing surgical planning and outcome prediction. Spectral imaging and near-infrared spectroscopy successfully quantified oxygenation and hemoglobin levels, offering reliable indicators of wound depth and healing potential. Spatial Frequency Domain Imaging (SFDI) provided additional insights by evaluating scar formation risk and potential infection. Furthermore, digital wound measurement tools, integrated with telemedicine platforms, supported precise, remote wound assessment and monitoring. Collectively, these imaging modalities contributed to a more comprehensive and effective approach to burn wound evaluation and management.

Nevertheless, each technique comes with its own limitations. While the MolecuLight^®^ autofluorescence device has demonstrated high sensitivity in detecting bacterial burden, the fluorescence signals emitted (particularly red) may derive not only from bacterial porphyrins but also from host tissue components, such as necrotic cells or inflammatory infiltrates. This can lead to false positives and reduced signal specificity. Additionally, technologies like, LDI, SFDI, and NIRS, while powerful, require skilled operators to generate and interpret results. Studies have demonstrated significant inter-user variability. In clinical settings with limited access to specialist training, these tools may yield inconsistent outcomes. Incorporating certified training programs or integrating AI-assisted interpretation may enhance reproducibility.

## 6. Discussion

The integration of imaging technologies into wound care represents a transformative advance in clinical practice. These modalities not only supplement traditional assessment techniques but also offer a new paradigm of precision, objectivity, and non-invasive diagnostics. The following sections will narrate through these technologies, highlighting their principles, clinical applications, and contributions to advancing wound care outcomes.

### 6.1. An Overview of Imaging Technology

#### 6.1.1. Optical Imaging Technologies

Some of the technologies that allow for an objective approach to wound assessment leverage light emission with pre-determined properties (specific wavelength, for instance) that measure the amount of light that is absorbed, scattered, or reflected back to generate useful information in relation to vascularity, perfusion, oxygenation, hemoglobin, and water levels, as well as collagen status [24].

##### Spectral Imaging

In spectroscopy imaging, capturing the reflected light from structures such as hemoglobin, deoxyhemoglobin, and melanin provides anatomical tissue mapping that elucidates the wound depth and oxygenation levels [25,26]. At a certain range of wavelengths, the spectroscopy device can depict the “chromophore of interest” (i.e., the hemoglobin, deoxyhemoglobin, and melanin) on a camera by assimilating and filtering the reflected light at various wavelengths. Each pixel in the resultant image corresponds to an absorption spectrum. By determining the extinction coefficients of that particular structural component of the skin (i.e., a chromophore), and also considering the intra-cutaneous travel length of light before reflection, the concentration of that particular chromophore can be estimated [27,28]. This modality has been applied in the assessment of burn wounds and diabetic ulcers. It has provided useful information in terms of depth measurement, particularly for burn wounds, and in the prediction of ulceration risk/healing potentials for both wound types [29,30].

##### Near-Infrared Spectroscopy

**Figure 1:**
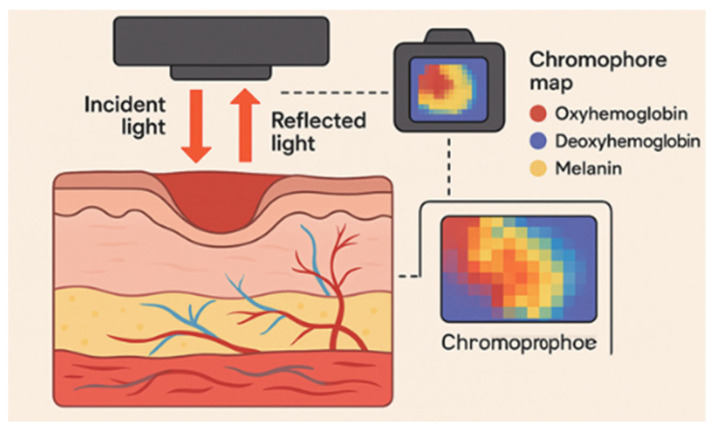
Near-infrared (NIR) spectroscopy imaging, illustration of the mechanism of action in wound assessment. Reflected light from hemoglobin, deoxy-hemoglobin, and melanin is filtered and analyzed to create anatomical tissue maps, aiding in the classification of wound depth and oxygenation levels for prognosis evaluation.

Other techniques adopt the same spectral principle. Near-infrared (NIR) spectroscopy identifies functional features like total hemoglobin and water content and structural tissues information and measures the reflected spectral signal to assess the burn depth changes [31,32,33]. Based on the intensity of the spectral signals, this type of technology aids in evaluating burn wound severity and in classifying burn wound depth (i.e., superficial to deep dermal and full-thickness burn wounds). This information enables the estimation of the healing prognosis in burn wounds and potentially others [32,34,35,36,37]. Figure 1 illustrates the mechanism of action of near-infrared (NIR) spectroscopy.

##### Orthogonal Polarization Spectral Imaging

**Figure 2:**
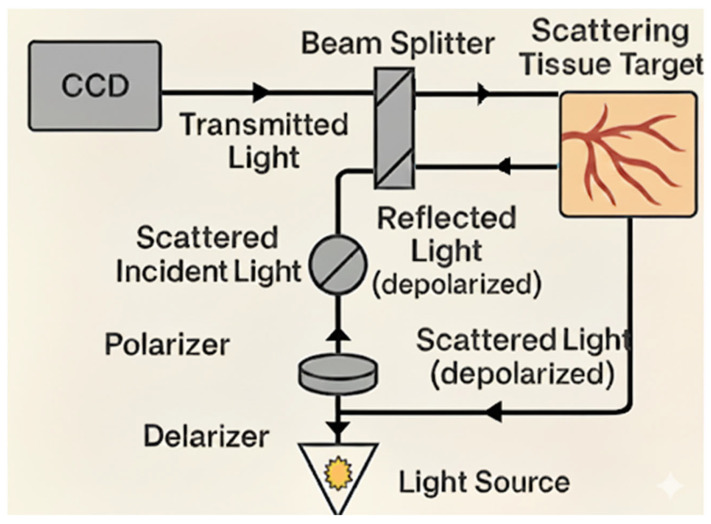
Orthogonal Polarization Spectral Imaging, illustration of the mechanism of action for burn depth classification. Polarized light at a specific wavelength is projected onto the tissue, penetrating deeply before becoming depolarized. An orthogonal polarizer eliminates unwanted reflections, allowing only deeply penetrating light to be captured by a charged coupled device (CCD) camera.

Another similar technology that can classify burn depth is Orthogonal Polarization Spectral Imaging (OPS). This modality also enables microvasculature assessment. In this technique, polarized light with a specific wavelength is projected to the tissue to be evaluated, and then the light is depolarized while penetrating deep into the tissue via targeting of the reflected light towards an orthogonal polarizer. Only the deeply penetrating lights will get depolarized, such that unwanted reflections are omitted. The reflected light is subsequently captured and recorded by a charge-coupled device (CCD) video camera. The polarizing light wavelength around (548 nm) is readily absorbed by hemoglobin. Hence, hemoglobin-containing erythrocytes will be visualized [38,39]. Figure 2 demonstrates the mechanism of action of this technology.

##### Spatial Frequency Domain Imaging

**Figure 3:**
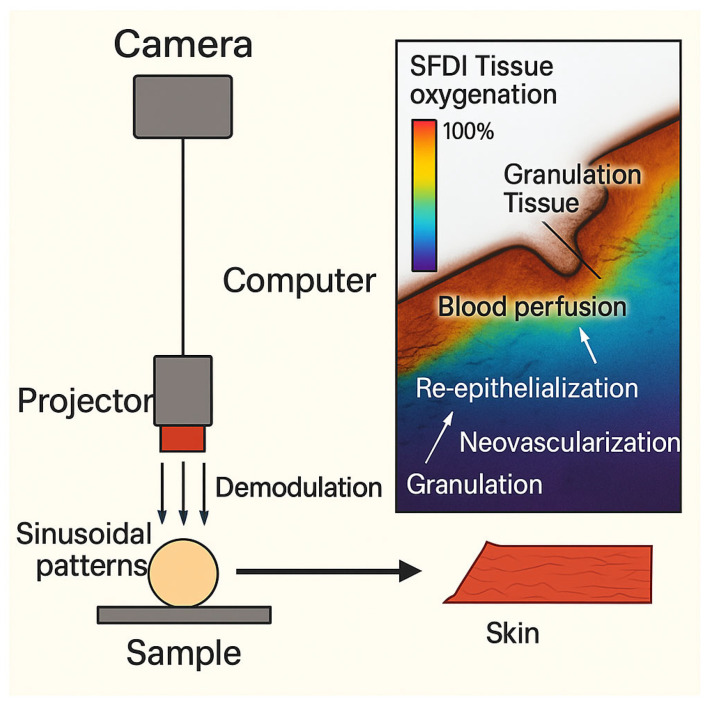
Spatial Frequency Domain Imaging (SFDI) for Wound Assessment. The SFDI setup used to project sinusoidal patterns onto a sample and acquire images. The resulting SFDI tissue oxygenation and blood perfusion map highlights various stages of wound healing.

Besides the burn wound depth, other clinical outcomes are of interest and can be identified in a proactive manner. Some examples are scar severity and early onset of burn wound infection. Other types of technologies can be employed for that purpose, for instance, Spatial Frequency Domain Imaging (SFDI), where a sinusoidal monochromatic light at a certain wavelength is deployed at three different phases onto the skin to be examined [40,41]. Light absorption and scattering are measured to infer the tissue vascularization and infection tendency. This is carried out by quantifying the degree of alternation in optical features like light absorption and reduced scattering, both of which usually occur as a result of bacterial infection [42]. Identifying such micro-physiological changes by SFDI is promising as an early identifier of vascular occlusion and proactive detection of flap loss [43,44,45,46]. Figure 3 illustrates the mode of action of this technique.

##### Laser Doppler Perfusion Imaging

Another non-invasive wound imaging method to assess tissue perfusion relies on a laser to determine microvascular blood flow [43,44,45,46]. Laser Doppler Perfusion Imaging (LDPI or LDI) captures the reflected change of the electromagnetic field scattered from examined tissues and mobile red blood cells (RBCs) after being illuminated with laser beams. Capturing the change in wavelength from moving RBCs reflects the erythrocytes’ velocity and quantity (i.e., tissue perfusion) [46,47]. This process has been used to assess burn wound severity [48,49]. Evidence shows that LDPI can more accurately assess the burn depth compared to visual clinical assessment and can precisely predict healing times [48,49,50], therefore enabling a prompt decision regarding graft timing as well as a better estimation of the hospital stay length with associated expenses [51,52]. LDPI is most efficacious in the postoperative evaluation of graft vascularization and in monitoring chronic skin ulcers [53,54,55,56,57].

#### 6.1.2. Thermal Imaging

Thermography is another non-invasive wound imaging device that works on the principle that all objects produce infrared signals that correspond to temperature readings and can be detected as color-coded thermal maps. Measuring temperature difference will reflect perfusion, hence, wound healing potentials. The skin’s surface can be classified on a thermographic basis according to the temperature it emits [58,59,60]. This principle has proven helpful in anticipating healing outcome and influencing management selection (e.g., whether spontaneous healing within 3–4 weeks is possible or if excision and grafting should be offered) [59,61,62].

#### 6.1.3. Fluorescence Imaging Techniques

##### Bacterial Fluorescence Imaging

**Figure 4:**
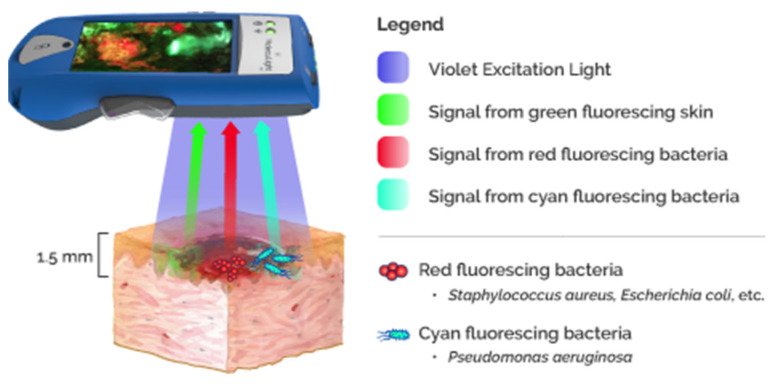
MolecuLight^®^ fluorescent imaging. The device and the principle of action.

Fluorescence Imaging (FI) leverages endogenous bacterial components found within bacterial species. The basic approach of the FI technique is to shine a safe 405 nm violet light to excite fluorophores in and around the wound up to a penetration depth of 1.5 mm [63]. Some fluorophores are specific to certain bacterial species; for instance, pyoverdine is an endogenous virulence factor of *Pseudomonas*, which enables the emission of a cyan fluorescent signal (REF) [64,65], and porphyrins are a byproduct of the heme pathway found in most bacterial species, which emit a red fluorescent signal visible under the violet light (REF) [65]. Other fluorescence imaging techniques require the use of exogenous fluorophores (“contrast agents”), which involve dye injection and subsequent assessment by non-invasive imaging techniques to assess vascular sufficiency and tissue perfusion [66]. These exogenous fluorescence techniques are considered impractical in some wound care settings; furthermore, the safety of these materials is still questionable in pediatrics and in pregnant or nursing women [67,68]. In contrast, the fluorescence imaging device (MolecuLight i:X, D:X, Toronto, ON, Canada) is safe and contact-free and captures fluorescent signals when bacterial concentrations are found at or surpass the 10^4^ CFU/gr of tissue threshold (REF) [69]. A specialized optical filter allows only the transmission of relevant fluorescence signals, maximizing their intensity by removing potential “noise”. The accuracy of these signals in the detection of moderate to high bacterial loads reported in literature ranges between 93% and 100% depending on the signals detected. The highest accuracy has been found with the cyan signal, which is specific for *Pseudomonas aeruginosa* (REF); however, the red signal can be emitted from other tissue components, such as necrotic cells or inflammatory infiltrates, resulting in false positives and reduced signal specificity [65,69,70]. It has been established in the literature that the MolecuLight^®^ bacterial fluorescence imaging device is a helpful tool to detect bacterial presence in real-time (at the bedside) and also enables targeted swabs and debridement [71,72,73,74,75]. The outcome-predicting capabilities of these signals have been proven, as positive bacterial fluorescence is directly linked to poor healing in chronic wounds, increased risk of infection and its complications, and the failure of CTPs (cellular tissue products, “skin substitutes”) (REFs) [76,77,78,79]. Figure 4 demonstrates the principle of MolecuLight^®^ action [13].

##### Fluorescence Lifetime Imaging

Nicotinamide Adenine Dinucleotide (NAD) is an example of an endogenous fluorophore that is used as a fluorescence source in the Fluorescence Lifetime Imaging (FLIM) technique [80]. In this technique, the light emitted for NAD excitation exhibits a marker for determining the skin healing process since NAD is generated in oxidative phosphorylation reactions and cellular metabolism. As a result, tissue viability and cutaneous healing can be measured [80].

#### 6.1.4. Digital Wound Measurement

Measuring wound dimensions is an essential factor in determining wound severity (i.e., classification), obtaining a baseline, monitoring healing progression, and determining subsequent interventions at different stages. The advent of digital imaging technologies makes three-dimensional, objective wound measurement possible. This is particularly important in complex wounds, when conventional, clinical visualization or two-dimensional images are inadequate to capture the extent of injury or the realistic correlation between the injured structures [81]. An example of this technique is stereophotogrammetry (SPG), formerly used in the field of land surveying [81]. Studies show that measuring wound dimensions by harnessing this technique has a high level of precision and provides more accurate information about wound depth and volume [82,83]. Since this technique is contactless, the risk of wound infection is limited.

Telemedicine plays a dramatic role in streamlining outpatient care flow and limiting infection spread while aiding in trimming costs and time consumption, which was particularly seen during the COVID-19 pandemic. The utility of SPG in remote wound assessment and diagnosis has been explored, with some limitations noted; most notably, the inaccurate determination of wound boundaries and other significant wound features like the presence of exudate and wound moisture level. These limitations emphasize the importance of integrating experts’ clinical observation with three-dimensional imaging [83,84].

Adding to the importance of aiming to increase the accuracy and objectivity of wound assessment processes, some technologies have incorporated features that improve workflow and ease of use. The MolecuLight^®^ imaging device, previously mentioned for its fluorescence imaging capabilities, includes a digital wound measurement feature that reportedly reaches over 95% accuracy (REF).

It is worth mentioning that, despite their promise, these technologies have limitations. For example, while MolecuLight^®^ can provide real-time infection detection, it cannot differentiate the culprit microorganism, apart from *Pseudomonas*, which can emit the specific cyan light; it also requires a dark environment to capture the image, which can be restricting, especially in operative settings [85]. Techniques like LDPI and SFDI require trained operators, and interpretation may be affected by patient movement or operator variability [86,87,88]. NIRS has limited penetration depth and is operator-dependent. Strategies to address these challenges include spectral unmixing, combining complementary imaging modalities, standardized training, and AI-assisted analysis to reduce variability and improve accuracy [89,90]. Additionally, thermal imaging is affected by the surrounding environment temperature and less accurate in partial vs. full-thickness differentiation, also, the OPS technique is less effective in carbonized/charring areas [91,92,93]. Direct, standardized comparisons between modalities are limited. While evidence suggests that LDPI remains the gold standard for burn depth assessment, SFDI provides complementary structural and functional information. Autofluorescence imaging is rapid for bacterial detection but lacks depth and perfusion data.

Future research should focus on large-scale, multi-center studies using standardized burn cohorts to test all various techniques and compare them with some outcome measures such as histopathology, healing time, and infection rates. Integrating imaging data with clinical findings could yield predictive models for individualized burn management, improve specificity in imaging, and enable broader access to operator-dependent modalities in resource-limited settings. Collectively, these imaging technologies are shifting wound care toward more objective, precise, and proactive management, offering opportunities to improve outcomes and personalize treatment strategies. Additionally, the adoption of advanced imaging technologies is strongly influenced by their high costs, which can range from tens to hundreds of thousands of pounds per device, depending on the modality and manufacturer. Systems like Spectral Imaging and SFDI require specialized equipment and software, while LDPI and NIRS devices, though somewhat more established, still represent a significant financial investment. These substantial costs act as a limiting factor in widespread availability, often restricting use to larger tertiary burn centers and contributing to a gradual, selective rollout rather than immediate, universal implementation across all units.

### 6.2. UK-Based Guidelines and Cost-Effectiveness

#### 6.2.1. MoorLDI2-BI NICE Innovation Briefing

**Figure 5:**
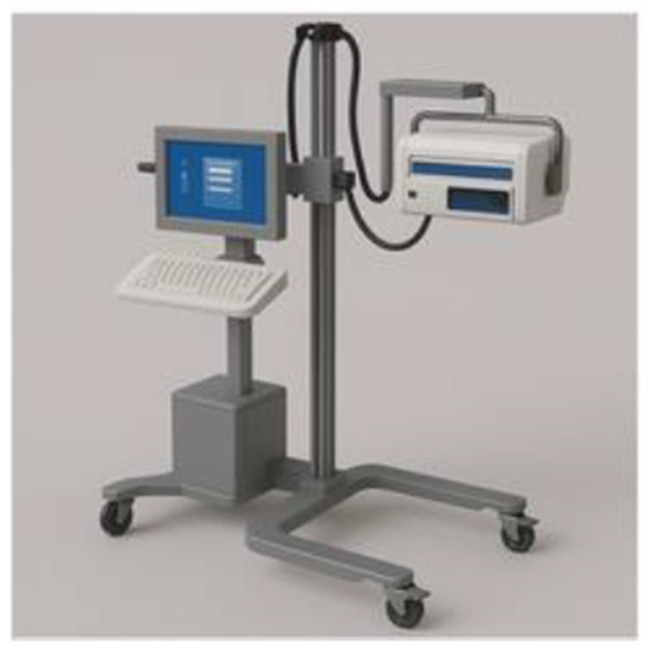
Schematic drawing of Moor LDI device.

In 2017, the National Institute for Health and Care Excellence (NICE) established a medical technology innovation. The featured outcome of deploying moorLDI2-BI was to formulate an improved care plan after accurately assessing the wound depth and to provide a prediction of the wound healing trajectory. Secondary utility outcomes included evading unnecessary interventions, lowering the frequency of dressing changes, and reducing the length of hospital stay. Longer-term outcomes can be measured by the extent of reclaiming pre-injury wound function and by studying the shape and extent of the resultant scar [86]. The NICE committee affirmed that there is sufficient evidence to support the routine use of the moorLDI2-BI device in the assessment of burns. The device is beneficial for anticipating wound healing direction, hence, shaping the subsequent surgical planning. This will also allow precocious surgical intervention, in certain cases, which may limit the extent of surgery. Precise demarcation of the wound needing to be grafted, for instance, will reduce the need for extensive excision [86]. Cost-wise, the analysis of the base case submitted by the manufacturers proposed a cost saving of GBP 1254 when the device is purchased, and GBP 1270 when it is leased, per patient, per operative burn wound treatment. Further savings could be attained by avoiding hospital admission altogether. In conclusion, the NICE committee approved moorLDI2-BI as a beneficial technique to support clinical decisions regarding burn depth and healing potentials in situations of diagnostic uncertainty, along with enhancing cost savings [86]. Figure 5 shows a schematic drawing of the Moor LDI device.

#### 6.2.2. MolecuLight NICE Innovation Briefing

Another medical technology that has undergone an innovation briefing published by NICE (2020) [94] is the MolecuLight i:X and its utility in diagnosing wound infection. As discussed, MolecuLight i:X is a handheld imaging device that can locate bacterial loads and biofilm in and around wounds and can also capture digital wound measurements.

Data from 177 adults and 10 children in secondary care and outpatient settings were utilized to conclude this briefing. The results demonstrated that MolecuLight i:X was able to identify wound bacteria at a comparable level to microbiology swabs. Wider scale multi-center Randomized Controlled Trials (RCTs) were suggested comparing the utility of MolecuLight i:X against the standard care to derive robust evidence, as the initial briefing was based only on observational studies with a limited sample size. A number of clinical studies, including an RCT, have been subsequently published. Results from a prospective, single-blind, multi-center cross-sectional study including 350 various wound types elucidated the superiority of MolecuLight i:X in terms of bacterial detection over the current diagnostic standard [69]. Results from the RCT (2022) [79] showed the utility of incorporating this device into standard wound care workup in terms of reshaping the fate of challenging, non-healing wounds and placing them back onto the healing pathway by early identification and rapid switching to targeted intervention. The study showed a doubling of diabetic foot ulcer healing rate at 12 weeks (22% versus 45%) when fluorescence imaging information was used to guide treatment in the intervention study arm [79]. Once the NICE guidelines are re-visited to include more recently published studies, we suspect support of this diagnostic intervention will be strengthened, and it could become the standard of care bedside diagnostic tool.

Finally, potential cost savings could also be gained by the early detection of wound infection. Prompt treatment may reduce complication rates and hospital stay duration. The technology also decreases the need for swabbing with its associated indirect expenditure (workforce load, for example), in addition to guiding graft timing and mitigating the expenses related to graft failure [85]. Further detailed cost-effectiveness analysis would pave the way for widespread adoption of this technology.

It is worth mentioning that cost-effectiveness evaluation in medical devices must account not only for the initial purchase price but also for operational logistics, including training, time, and staffing. For example, the MolecuLight device, which costs around GBP 25,000, can lead to savings by reducing infection-related hospital stays. Laser Doppler Imaging (LDI), with a higher cost of over GBP 45,000, offers long-term savings through more accurate surgical planning. The SPG device, with a more moderate upfront cost, proves valuable particularly in outpatient settings and telemedicine. However, high initial investment and the need for specialized training may limit their affordability. Additionally, inconsistent clinical evidence or a lack of standardization can hinder widespread application of trust. Table S1 summarizes the mode of action, clinical applications, diagnostic accuracy metrics, and limitations of key imaging technologies discussed, providing a comparative perspective for their integration into clinical practice.

### 6.3. Emerging Imaging Technologies

Examples of emerging techniques include Optical Coherence Tomography (OCT), which is a non-invasive imaging technique that uses near-infrared light to generate cross-sectional images of skin layers, enabling detailed assessment of burn severity, healing progression, and surgical margins. However, OCT has limitations, including shallow tissue penetration (only 1–2 mm), a small field of view that restricts large area imaging, high equipment costs, and the need for trained personnel, making it less accessible in low-resource settings [95]. AI-assisted imaging leverages machine learning algorithms to classify burn depth, segment affected areas, and predict treatment outcomes with high accuracy, reducing dependence on clinical judgment. Yet, data governance issues, like privacy and confidentiality, are not fully addressed. Multispectral Imaging (MSI), which captures data across multiple wavelengths, provides critical insights into tissue perfusion and oxygenation, assisting in burn depth assessment, intraoperative guidance, and wound monitoring. Despite its portability and non-invasiveness, MSI is sensitive to motion artifacts and environmental conditions, offers limited depth resolution, and requires complex data interpretation tools. Meanwhile, 3D imaging creates volumetric reconstructions of burn wounds, allowing for precise measurement of wound geometry, volume, and surface characteristics, which are essential for surgical planning and healing assessment. Still, it only captures surface data without depth insight, is costly, and involves time-intensive processing and technical demands [96]. Each of these technologies brings unique advantages, but their limitations must be carefully considered to ensure appropriate application in diverse clinical settings.

In major UK burn centers, several advanced imaging technologies are being integrated into clinical practice. Spectral Imaging, such as the DeepView™ System by Spectral MD (Dallas, TX, USA) has received UKCA authorization for burn indications, with six devices expected to be deployed across centers for evaluation and clinical use [97]. LDPI is a well-established technique for evaluating microvascular blood flow and tissue perfusion, and it is readily available in the major UK burn centers, attributed to its role in assessing burn depth and predicting healing outcomes. MolecuLight i:X was introduced to the UK in March 2018, with 100 devices used across the NHS and private healthcare. Collectively, these technologies are progressively being incorporated into clinical practice in the UK to improve the accuracy of burn assessments and enhance patient outcomes. However, their high cost limits their availability, restricting use primarily to major burn centers.

## 7. Conclusions

The domain of wound imaging is rapidly progressing. While some techniques are in their experimental stage, other modalities proceed to full translation into daily clinical work. Evidence holds some positive promises of integrating imaging technology into the current practice. However, full incorporation has yet to occur for most imaging procedures. The potential positive economic impact is also tangible. The future should open avenues for more research to extensively explore and validate those findings.

### Key Findings

- MolecuLight Fluorescence Imaging detected bacterial burden with over 90% accuracy and enabled real-time wound measurement.
- Laser Doppler Imaging (LDI) accurately assessed tissue perfusion and burn depth, improving surgical planning and outcome prediction.
- Spectral Imaging and Near-Infrared Spectroscopy quantified oxygenation and hemoglobin levels, aiding in depth assessment and healing prediction.
- Spatial Frequency Domain Imaging (SFDI) evaluated scar formation risk and potential infection.
- Digital Wound Measurement and Telemedicine enhanced remote assessment and ensured precise, ongoing wound monitoring.
- Combined, these technologies provided a comprehensive, non-invasive approach to burn wound evaluation and management.

## Supplementary Materials

The following supporting information can be downloaded at https://www.mdpi.com/article/10.3390/diagnostics15172277/s1, Table S1: Comparative Performance of Advanced Imaging Technologies for Burn Assessment.
