# Supplementary material for: How Can Technology Improve Burn Wound Care: A Review of Wound Imaging Technologies and Their Application in Burns—UK Experience
Source: Diagnostics (Basel). 2025 Sep 8;15(17):2277. doi: 10.3390/diagnostics15172277 (PMC12427859; doi:10.3390/diagnostics15172277)
Supplement: Supplementary file 1 [file diagnostics-15-02277-s001.zip › diagnostics-3752655-supplementary.pdf]

Table (S1): Comparative Performance of Advanced Imaging Technologies for Burn Assessment

Imaging Technology [Reference]	Mode of Action	Burn-Specific Indications	Sensitivity (%)	Specificity (%)	Positive Predictive Value (%)	Negative Predictive Value (%)	Burn-Specific Limitations
Spectral Imaging [28,49]	Captures reflected light at multiple wavelengths to map chromophore distribution (e.g., hemoglobin, melanin) and infer tissue oxygenation and depth.	Differentiates burn depth; assesses tissue oxygenation and perfusion to guide surgical timing.	85–95	80–90	88–92	85–90	Reduced accuracy in highly pigmented skin; limited penetration in thick eschar.
Near-Infrared Spectroscopy [89,90]	Measures absorption of near-infrared light by hemoglobin and water to estimate tissue oxygenation and hydration.	Classifies burn depth (superficial, deep dermal, full-thickness); predicts healing potential.	90–95	85–90	90–93	88–92	Reduced accuracy in deep dermal/full-thickness burns; affected by edema, skin thickness.

Orthogonal Polarization Spectral Imaging [34,93]	Uses polarized light at ~548 nm to visualize microvasculature by filtering depolarized light absorbed by hemoglobin.	Assesses microvascular integrity and burn depth; useful in borderline burn areas.	88–92	80–85	86–89	83–86	Requires expertise; less effective in carbonized/charring areas.
Spatial Frequency Domain Imaging [42,88]	Projects sinusoidal light patterns at multiple phases; measures light absorption and scattering to assess tissue structure and perfusion.	Early detection of infection or vascular compromise; adjunct for depth and scar prediction.	85–90	80–85	85–88	82–85	Motion-sensitive; infection signals may overlap with inflammation.
Laser Doppler Perfusion Imaging [86,87]	Uses low-power laser light to detect frequency shifts in backscattered light caused by moving red blood cells, estimating microvascular blood flow.	Gold standard for burn depth assessment; predicts healing and guides graft timing.	90–95	85–90	90–92	88–91	Requires patient stillness; difficult over joints or mobile areas.

Thermal Imaging [91,29]	Detects infrared radiation emitted by skin to create temperature maps, inferring perfusion and viability.	Differentiates viable vs. non-viable tissue; supports grafting decisions and healing prediction.	75–85	70–80	77–82	73–78	Influenced by ambient temperature; less accurate in partial vs. full-thickness differentiation.
Fluorescence Imaging [69,72,85]	Uses 405 nm violet light to excite bacterial fluorophores (e.g., porphyrins, pyoverdine); emits characteristic red or cyan fluorescence detected via optical filters.	Detects and localizes bacterial burden in burns; guides targeted debridement and swabbing; supports infection risk assessment.	93–100	85–90	94–96	92–95	Red signal can originate from host tissue (false positives); requires operator training; limited penetration depth (~1.5 mm).
